# Comparing postoperative complication of LigaSure Small Jaw instrument with clamp and tie method in thyroidectomy patients: a randomized controlled trial [IRCT2014010516077N1]

**DOI:** 10.1186/s12957-018-1448-9

**Published:** 2018-09-21

**Authors:** Ali Ramouz, Seyed Ziaeddin Rasihashemi, Abdolrasoul Safaeiyan, Mahdie Hosseini

**Affiliations:** 1 Department of Cardiothoracic Surgery, Tabriz University of Medical Sciences, Tabriz, Iran; 2 Department of Vital Statistics and Epidemiology, School of Health, Tabriz University of Medical Sciences, Tabriz, Iran; 3 Imam Reza Hospital, Golgasht street, 5183915881, Tabriz, Iran

**Keywords:** Thyroidectomy, LigaSure® Small Jaw, Conventional, Hypocalcemia, Recurrent laryngeal nerve

## Abstract

**Background:**

LigaSure® Small Jaw (LSJ) has been recently introduced as an energy-based vessel sealing device, which has provided better intraoperative and postoperative outcomes in thyroidectomies, compared to conventional technique. In the current study, we aimed to examine the efficiency of hand-sewn and LSJ thyroidectomy, based on operation time and perioperative complications.

**Methods:**

All patients with the diagnosis of multinodular goiter, thyroid cancers, retrosternal goiter and other indications for thyroid surgeries, enrolled. Of 550 patients, 261 patients randomly assigned to the conventional group (A) and 274 patients to LigaSure Small Jaw group (B). Study groups compared concerning operative time, recurrent laryngeal nerve (RLN) injury, hypocalcemia, and postoperative complications.

**Results:**

There was no significant difference regarding demographic data between groups A and B. During total thyroidectomy, intraoperative blood loss was 64.42 ± 20.72 ml and 49.64 ± 17.92 ml in groups A and B, respectively (*P* 0.043). Operative time was significantly lower in LSJ group compared to the conventional group in total and subtotal thyroidectomy (*P* 0.002; *P* 0.001). Three patients who underwent conventional total thyroidectomy had RLN palsy. However, there was no significant difference between techniques regarding RLN injury (*P* 0.134).

Postoperative total and ionized serum calcium levels decreased compared to preoperative levels in both conventional and LSJ technique; however, changes in total and ionized serum calcium were more severe in patients with conventional thyroidectomy (total calcium, *P* < 0.0001) (ionized calcium, *P* 0.005).

**Conclusion:**

The LigaSure Small Jaw device decreases operative time and intraoperative bleeding compared to conventional technique. Besides, changes in total and ionized calcium levels in patients with LSJ thyroidectomy are subtle compared to HS technique.

**Trial registration:**

Registered in Iranian Registry of Clinical Trials (www.irct.com), trial registration: IRCT2014010516077N1, Registered: 23 May 2014).

## Background

Before the nineteenth century, the thyroid gland surgeries were associated with dangerous complications due to its vascularization and adjacency with vital organs [1, 2]. Thus, reported complication rates in the published literature were up to 50%, with a mortality rate of 20% [3, 4]. However, during the first half of the twentieth century, Kocher introduced a new hemostasis technique consisting of suture ligation of vessels by clamp and tie method. After great development, complication rates after thyroid surgery decreased to 1% and lowered in experienced hands [5–7]. Although conventional ligation technique provides meticulous hemostasis and is still known as the gold standard approach for thyroid surgeries, its disadvantages, such as time-consuming vessel ligation, hematoma, transient or permanent recurrent laryngeal nerve (RLN) injury, and hypocalcemia, have led to several attempts to achieve a better technique [8, 9].

During recent years, numerous methods for vessel ligation have been developed leading to reduce operation time and further complications. One of the most advanced surgical devices, LigaSure® Small Jaw (Covidien, Boulder, CO, USA) is an energy-based device that provides a rapid vessel sealing system (VSS), capable to dissect, ligate, and cut the vessels with a diameter up to 7 mm. Also, LSJ has a unique design that prevents electrical power transmission and heat transfer leading to severe complications [8, 10].

In a study by Molnar et al. on 20 patients, they showed significantly lower operation time for total thyroidectomy, while using LSJ [10]. However, despite the extensive use of new generation of LigaSure vessel sealing system, few studies have compared its advantages with other techniques and surgical devices; therefore, LSJ priority is still controversial. In the current single-blinded randomized controlled trial, we aimed to examine the efficiency of conventional and LSJ thyroidectomy, based on operation time, and perioperative complications including bleeding, RLN injury, hematoma, and hypocalcemia.

## Methods

### Patients

**Fig. 1:**
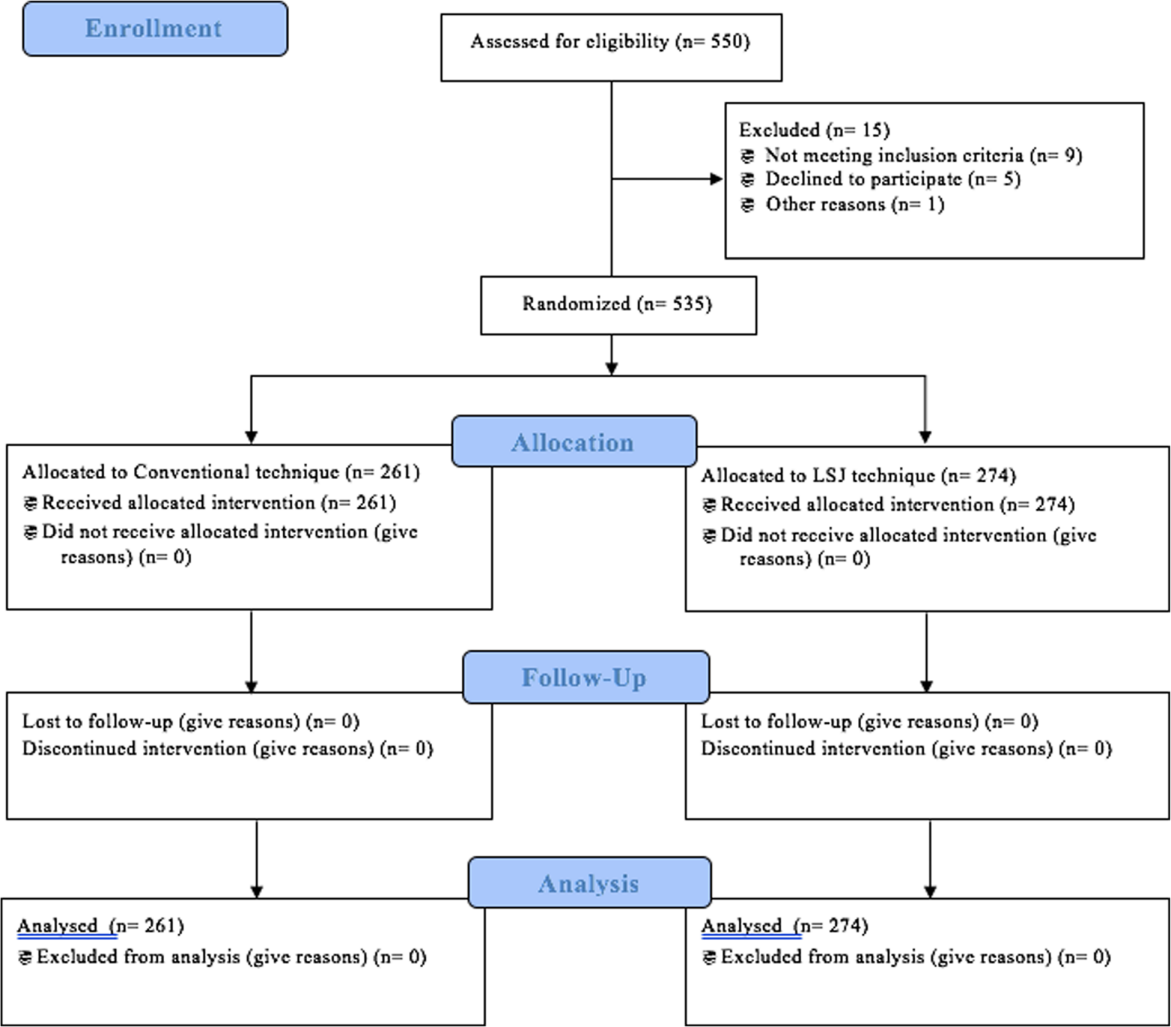
CONSORT 2010 study flow diagram

A single-blinded, two-central randomized controlled trial was carried out between May 2014 and March 2016, in thoracic and general surgery wards, Imam Reza and Taleghani hospitals, Tabriz University of Medical Sciences. The study was approved by the ethics committee of the Vice Chancellor of Research and Development, Tabriz University of Medical Sciences (Committee reference number: 92170), and registered in Iranian Registry of Clinical Trials (www.irct.com), Trial registration: IRCT2014010516077N1, Registered: 23 May 2014). All patients with diagnosis of multinodular goiter, thyroid cancers, retrosternal goiter, and other indications for thyroid surgeries were enrolled. Patients who had undergone neck surgeries or were candidate for secondary thyroidectomies, patients suffering underlying parathyroid diseases and who take calcium supplements, and patients whose surgeons failed to preserve parathyroid glands during surgery were excluded. All patients provided written informed consent to participate in the study. Of 550 patients (120 patients, candidate for subtotal thyroidectomy; and 430 patients, candidate for total thyroidectomy), 15 patients were excluded and 535 patients randomly assigned to study groups. Of 15 excluded patients, four patients had hyperparathyroidism, three patients consumed calcium supplements, one patient suffered unintended parathyroidectomy, one patient had a previous history of neck surgery due to trauma, five patients declined to participate in the study, and one patient died before randomization. Study groups were categorized as follows: (A) patients who underwent thyroidectomy by conventional technique including classic clamp and tie vessel ligature (hand-sewn technique (HS)) and (B) patients who underwent thyroidectomy using LigaSure Small Jaw to achieve hemostasis (LigaSure Small Jaw technique (LSJ)) (Fig. 1). Randomization was performed using a computer via a statistician who was blinded to the patient data and surgery technique.

Demographic data, including patients’ age, gender, body mass index (BMI), postoperative histopathology study, were recorded. All patients underwent preoperative physical examination and laboratory tests including total and ionized calcium level, prior to surgery. During postoperative admission, patients were examined to determine symptomatic hypocalcemia. Before parathyroid glands manipulation, total and ionized calcium levels measured 24–48 h and 2 weeks after surgery. Intraoperative blood loss was calculated simultaneously and meticulously by an anesthetic team with due attention to suction canisters and surgical sponge weighting.

Patients, suffering hypocalcemia symptoms with a total calcium level less than 8.5 mg/dl or an ionized calcium level less than 1.0 mmol/l, received oral or intravenous repletion based on hypocalcemia severity.

Also, considering the probability of transient superior laryngeal nerve (SLN) injury due to its adjacency to the superior pole of the thyroid gland, patients underwent an examination to evaluate SLN injury. Since there was a lack of the gold standard diagnostic method for SLN injury determination, we considered reduced pitch and poor-quality voice as symptoms of SLN injury, which evaluated by two independent, experienced blinded otolaryngologists.

### Surgical procedure

All surgical procedures were performed by the same surgeon specialized in thyroid surgery. We used the standard operative technique in both groups for thyroidectomy. A 3–5-cm curvilinear collar-type incision was placed 1 cm below cricothyroid cartilage. The subcutaneous layer and the platysma muscle were cut, and subsequently superior and inferior subplatysmal flaps were raised up to the superior border of thyroid cartilage and sternal notch, respectively. In LSJ group including 261 patients, we used the LigaSure Small Jaw device for ligation superior, middle and inferior thyroid vessels individually. LSJ was used for dissecting the dense attachments at the level of the posterior suspensory ligament (Berry), to release the isthmus attached to the trachea while preserving the RLN and the parathyroid glands. In case of failure to protect parathyroid glands, patients were excluded from the study group postoperatively.

The operative technique in HS group was conventional clamp-tie technique using classic devices.

Like monopolar electrocautery, absorbable and non-absorbable suture material for vascular ligatures. After extubating in the operation room, patients underwent direct laryngoscopy to evaluate vocal cords and RLN function.

### Statistical analysis

We calculated the study power considering 535 patients sample size needed to compare two means: two-sample and one-sided. Amount of the blood loss during total thyroidectomies recognized as the primary endpoint of the study and mean amount in groups (A) and (B) were 64.42 ± 20.72 ml and 49.64 ± 17.92 ml, respectively. Considering sample size of 535 patients, type I error rate (α) of 0.05, and sampling ratio of 1.04, the study power (1-β) was calculated to be more than 95%. The influential variables were adjusted between two intervention groups (manual and mechanical) at the beginning of the study. For quantitative data, normality was evaluated by K-S test, and then Mauchly’s *W* test was checked for identity covariance matrix, and finally repeated measure with control covariates test was used by Minitab Software version 17. The results include six *P* values for comparing groups between different times. The first was *P* value matching for comparing variations before the beginning intervention groups, and the second was *P* value. Treatment for differentiating between two intervention groups (manual and mechanical) is the final *P* value time which is used for comparing each variable between before and after the intervention.

The chi-square test was used for comparing two qualitative variables in each time and Cochran’s *Q* for comparing dependent variables between different times. The level of significance was set at 0.05, and all results were expressed by frequency (percent) for qualitative variables and mean ± SE for quantitative variables.

## Results

Patients’ demographic data are listed in Table 1, according to surgery type and technique. Considering patients’ age, gender and thyroid pathology, there were no significant differences between LSJ and HS groups. Also, there were no significant differences between study groups about preoperative ionized and total serum calcium levels, in both total and subtotal thyroidectomies.

**Table 1:** Patients’ demographic data and characteristics

Variable	Technique		*P*
	HS group (*n* 273)	LSJ group (*n* 262)	
Age	45.04	44.94	0.62
Gender			
Male	49 (18.8%)	61 (22.3%)	0.18
Female	212 (81.2%)	213 (77.7%)	
BMI (kg/m2)	26.4	26.3	0.47
Thyroidectomy			
Subtotal	50	60	0.24
Total	211	214	
Thyroid pathology			
MNG	119 (43.4%)	137 (52.5%)	0.29
Retrosternal MNG	23 (8.4%)	15 (5.7%)	
PTC	73 (26.6%)	65 (24.9%)	
FTC	15 (5.5%)	8 (3.1%)	
MTC	12 (4.4%)	4 (1.5%)	
HCC	5 (1.8%)	9 (3.4%)	
Thyroid adenoma	10 (3.6%)	8 (3.1%)	
Grave’s	5 (1.8%)	5 (1.9%)	
Nodule	5 (1.8%)	4 (1.5%)	
Hashimato	4 (1.5%)	2 (0.8%)	
Cyst	2 (0.7%)	4 (1.5%)	

*Abbreviations: HS* hand Sewing, *LSJ* LigaSure Small Jaw, *BMI* body mass index, *MNG* multinodular goiter, *PTC* papillary thyroid carcinoma, *FTC* follicular thyroid carcinoma, *MTC* medullary thyroid carcinoma, *HCC* Hurthle cell carcinoma

Intraoperative variables including the amount of bleeding and operation time are summarized in Table 2. Although our results showed significant reductions in intraoperative blood loss during total thyroidectomy in LSJ group (*P* 0.04), there was no significant difference between the two groups, during subtotal thyroidectomy (*P* 0.06). However, mean operative times were significantly lower in the LSJ group compared to the HS group, during both total and subtotal thyroidectomies (*P* < 0.01; *P* < 0.01).

**Table 2:** Intraoperative variables and postoperative complications

	Technique		*P*
	HS group (*n* 274)	LSJ group (*n* 261)	
Blood loss (mm)			
Total thyroidectomy	49.64 ± 17.92	64.42 ± 20.72	0.04
Subtotal thyroidectomy	20.77 ± 14.55	35.07 ± 18.78	0.06
Operation time (mm)			
Total thyroidectomy	153.45 ± 43.34	97.34 ± 31.68	< 0.01
Subtotal thyroidectomy	66.46 ± 23.62	45.37 ± 12.30	< 0.01
RLN palsy			
Total thyroidectomy	3 (1.09%)	0	0.13
Subtotal thyroidectomy	0	0	–
Transient SLN injury	9 (3.2%)	17 (6.5%)	0.03
Hematoma	6 (2.1%)	2 (0.7%)	0.11
SSI	5 (1.8%)	2 (0.7%)	0.22
Symptomatic hypocalcemia			
Total thyroidectomy	27 (12.6%)	17 (8.1%)	0.03
Subtotal thyroidectomy	15 (25%)	6 (12%)	0.04
Permanent hypocalcemia	1 (0.3%)	2 (0.7%)	0.47

*Abbreviations: HS* hand sewing, *LSJ* LigaSure Small Jaw, *RLN* recurrent laryngeal nerve, *SLN* superior laryngeal nerve, *SSI* surgery site infection

Postoperative variables, including RLN function, external branch of SLN injury, hematoma formation, surgery site infection (SSI), and symptomatic hypocalcemia, were summarized in Table 2. Three patients (0.5%) who underwent total thyroidectomy by HS technique had unilateral RLN palsy (two right RLN and one left RLN). However, there were no significant differences between two methods (*P* 0.12). Transient SLN injury was detected in 26 patients (4.8%), whereas for the LSJ group, there was significantly associated higher prevalence of SLN injury compared to HS group (*P* 0.04). Symptomatic hypocalcemia was obtained in 65 (12.1%) patients, who had the complaint of numbness or tingling in fingers and toes or were positive for Chvostek and Trousseau signs. Patients who underwent LSJ total thyroidectomy had significantly less prevalence of symptomatic hypocalcemia compared to HS group (*P* 0.03). Additionally, during subtotal thyroidectomy, symptomatic hypocalcemia was substantially more prevalent in the HS group (*P* 0.04). Permanent hypocalcemia was observed in three patients (0.5%), which showed no significant difference between HS and LSJ groups (*P* 0.47). While comparing postoperative hematoma formation and SSI, there were no significant differences between study groups. Mean hospital stay of patients is given in Table 2. However, our study revealed no significant difference between study groups.

Total and ionized serum calcium levels are summarized in Table 3. We compared the pattern of changes in calcium levels, after thyroidectomy, to evaluate the technique effect on parathyroid gland manipulation and postoperative hypocalcemia. The results showed without considering thyroidectomy type, postoperative total and ionized serum calcium levels decreased compared to preoperative levels in both HS and LSJ technique. However, changes in total and ionized serum calcium levels in patients who underwent HS thyroidectomy were more severe than LSJ group (total calcium, *P* < 0.01) (ionized calcium, *P* < 0.01).

**Table 3:** Pre- and postoperative total and ionized calcium in HS and LSJ groups

Variable		Thyroidectomy	Subtotal			Total			*P*			Machine
		Technique	HS group	LSJ group	Independent *t* test	HS group	LSJ group	Independent *t* test	Mixed model			
		Time	Mean ± S.D.			Mean ± S.D.			Surgery type	Interaction	Time	
Calcium	Total (mg/dl)	Day before surgery	9.89 ± 0.86	9.88 ± 0.89	0.47	9.87 ± 1.02	9.89 ± 0.92	0.57	< 0.01	0.01	< 0.01	0.91
		Day after surgery	9.01 ± 0.94	9.45 ± 1.12	0.39	8.81 ± 0.74	8.98 ± 0.82	0.1				
		2 weeks after surgery	9.11 ± 0.92	9.55 ± 0.10	0.85	9.21 ± 0.97	9.21 ± 1.06	0.1				
	Ionized (mmo/l)	Day before surgery	1.09 ± 0.23	1.09 ± 0.12	0.62	1.091	1.088	0.07	0.01	0.65	< 0.01	0.98
		Day after surgery	1.03 ± 0.18	1.05 ± 0.33	0.08	1.019	1.031	0.09				
		2 weeks after surgery	1.01 ± 0.19	1.06 ± 0.13	0.03	1.016	1.055	< 0.01				

*Abbreviations: SD* standard deviation, *HS* hand sewing, *LSJ* LigaSure Small Jaw

## Discussion

Thyroid surgery has been widely increased with advances in surgical techniques, becoming the most common endocrine surgery [3, 11]. However, prolonged operative time and high demand for surgical skills are severe weaknesses of the conventional technique [8, 12, 13]. The LigaSure Small Jaw is a novel instrument that provides excellent hemostasis while decreasing thermal injuries compared with previous energy-based devices [14]. Therefore, due to thyroid abundant blood supply and anatomical adjacency to vital organs, LSJ vessel sealing system has been employed in thyroid surgery to minimize undesirable vascular and nerve damage [15]. Unlike conventional thyroidectomy, the electrical vessel sealing system has been suggested to reduce the time of surgery, RLN damage, and parathyroid manipulation [10, 16].

In the current randomized controlled trial, results showed a significantly higher prevalence of subclinical and clinical hypocalcemia after conventional thyroidectomy. To our knowledge, it was the first study to compare post-thyroidectomy hypocalcemia prevalence between LSJ and hand-sewn technique, regarding total and ionized calcium levels changing the pattern, during 2-week postoperative. In a study conducted on patients with papillary thyroid carcinoma, there was no significant difference concerning postoperative complications between the harmonic scalpel and LSJ instruments. However, ionized serum calcium was significantly higher in patients underwent LSJ thyroidectomy [17]. We analyzed the pattern of changes in serum total and ionized calcium levels postoperatively that revealed the fewer scope of changes in LSJ group. In a study, temporary hypoparathyroidism following LSJ thyroidectomy was reported to be lower than conventional technique [13]. Similarly, we found a lower rate of symptomatic hypocalcemia after LSJ total and subtotal thyroidectomy. However, three patients (one in HS group and two in LSJ group) suffered permanent hypocalcemia and received long-term calcium and vitamin D supplement. The role of the old generation of LigaSure in reducing rate of postoperative hypocalcemia is controversial; however, along with our study, some studies reported significantly lower rate of hypocalcemia using a new generation of LigaSure Small Jaw [2, 18–20].

In the present study, operative times were significantly lower in patients who underwent LSJ total and subtotal thyroidectomy with a mean reduction of 56 and 19 min, respectively. However, in a randomized controlled trial conducted on patients with a solitary thyroid nodule, the mean decrease in operative time was 21 min in LSJ group comparing to the conventional group [21]. In contrary, Hammad et al. showed no significant difference concerning operative time between mechanical and hand-sewn methods [22], which the difference may be due to variations in the study type and design.

Overall, recurrent laryngeal nerve palsy was observed in 1% of the patients who underwent total thyroidectomy and not detected in patients with subtotal thyroidectomy. However, there was no significant difference between LSJ and HS thyroidectomies [21]. Despite the fact that LSJ improves the ability of the surgeon to reduce the side effects of manipulation, however, complications after conventional thyroid surgery depends on the surgeon’s experience.

LigaSure Small Jaw instrument has been reported to decrease lateral thermal injury due to unique design [13]. However, in the present study, we observed that prevalence of the external branch of the SLN injury was significantly higher in patients underwent LSJ thyroidectomy. Nonetheless, all patients with voice problems caused by SLN injury improved within 2 weeks follow-up.

Regarding intraoperative blood loss during total and subtotal thyroidectomies, patients underwent thyroidectomy using LSJ had significantly less bleeding, which was in contrary with the previous study on LigaSure [19]. Our results showed no significant differences regarding hematoma and SSI prevalence among LSJ and HS groups. However, of four patients who suffered postoperative hematoma, hematoma was resolved in three patients after serial fluid aspiration and one patient underwent reoperation to acquire hemostasis and long-term complications developed in none of the patients, similar to the previous studies [23]. There was no significant difference concerning hospital stay between LSJ and HS groups.

We believe our study has some limitations. First, all surgeries including LSJ and conventional technique was performed by a single surgeon. However, the surgeon had approximately same experience both on conventional and LSJ techniques. Second, similar to previous studies, the included surgeon was not blinded to patients’ allocated study group. Third, due to a significant difference between hospital and devices costs in our country and developed countries, we were unable to compare prices between LSJ and conventional technique. Finally, despite the large population of the study, still, we were unable to detect prevalence and incidence of some of the rare complications, such as RLN injury and permanent hypocalcemia, through present study.

Our results showed that LigaSure Small Jaw device is an excellent alternative instrument for the suture-ligation procedure in thyroid surgery about decreased operative time and complication rate. Besides, since the majority of our patients were suffering thyroid cancer with different histopathology, it can be estimated that LSJ instrument provides better intraoperative and postoperative complications in thyroid cancer patients who are candidate for total and subtotal thyroidectomy.

## Conclusion

The LigaSure Small Jaw device decreases operative time and intraoperative bleeding compared to conventional technique. Also, changes in total and ionized calcium levels in patients with LSJ thyroidectomy is subtle compared to HS technique. However, there was no significant difference between the two methods regarding RLN injury and hematoma formation.
